# Effect of Ion and Binding Site on the Conformation of Chosen Glycosaminoglycans at the Albumin Surface

**DOI:** 10.3390/e24060811

**Published:** 2022-06-10

**Authors:** Piotr Sionkowski, Piotr Bełdowski, Natalia Kruszewska, Piotr Weber, Beata Marciniak, Krzysztof Domino

**Affiliations:** 1 Institute of Theoretical and Applied Informatics, Polish Academy of Sciences, ul. Bałtycka 5, 44-100 Gliwice, Poland; piotr.sionkowski@gmail.com (P.S.); kdomino@iitis.pl (K.D.); 2 Institute of Mathematics and Physics, Bydgoszcz University of Science and Technology, 85-796 Bydgoszcz, Poland; nkruszewska@pbs.edu.pl; 3 Institute of Physics and Applied Computer Science, Faculty of Applied Physics and Mathematics, Gdańsk University of Technology, ul. G. Narutowicza 11/12, 80-233 Gdańsk, Poland; piotr.weber@pg.edu.pl; 4 Faculty of Telecommunications, Computer Science and Electrical Engineering, Bydgoszcz University of Science and Technology, 85-796 Bydgoszcz, Poland; beata.marciniak@pbs.edu.pl

**Keywords:** human serum albumin, hyaluronan, conformational entropy, dihedral angles, frequency distribution

## Abstract

Albumin is one of the major components of synovial fluid. Due to its negative surface charge, it plays an essential role in many physiological processes, including the ability to form molecular complexes. In addition, glycosaminoglycans such as hyaluronic acid and chondroitin sulfate are crucial components of synovial fluid involved in the boundary lubrication regime. This study presents the influence of Na${}^{+}$, Mg${}^{2+}$ and Ca${}^{2+}$ ions on human serum albumin–hyaluronan/chondroitin-6-sulfate interactions examined using molecular docking followed by molecular dynamics simulations. We analyze chosen glycosaminoglycans binding by employing a conformational entropy approach. In addition, several protein–polymer complexes have been studied to check how the binding site and presence of ions influence affinity. The presence of divalent cations contributes to the decrease of conformational entropy near carboxyl and sulfate groups. This observation can indicate the higher affinity between glycosaminoglycans and albumin. Moreover, domains IIIA and IIIB of albumin have the highest affinity as those are two domains that show a positive net charge that allows for binding with negatively charged glycosaminoglycans. Finally, in discussion, we suggest some research path to find particular features that would carry information about the dynamics of the particular type of polymers or ions.

## 1. Introduction

Lubrication in natural joints is a complex multiscale process that involves interactions between constituents of articular cartilage and synovial fluid [1,2,3]. Although two main mechanisms—hydration repulsion and molecular synergies—have been found, the atomistic details on phenomena are still studied due to the lack of knowledge of how the cooperation between them results in facilitated lubrication [4,5,6,7,8]. Synovial fluid consists of up to 80% of water, macromolecular and small-molecular components.

The most recognizable components are: albumin, hyaluronan, phospholipids, γ-globulin and lubricin [9]. Human serum albumin (HSA) deserves special attention due to its binding and transporting properties of various compounds (fatty acids, ions: K${}^{+}$, Na${}^{+}$, Mg${}^{2+}$, Ca${}^{2+}$ and many other molecules [10,11]). It was demonstrated in [12,13] that albumin and γ-globulin play an important role in lubrication. However, their influence on lubricating properties starts to be vital when taking into account their cooperation with other synovial fluid components. Locally positively charged sites of albumin favor interactions with the ionized carboxylic and sulfate groups in glycosaminoglycans (GAGs). Even though both macromolecules have a global negative charge under physiological conditions, it was shown that HSA could bind to negatively charged surfaces [14].

**Figure 1:**
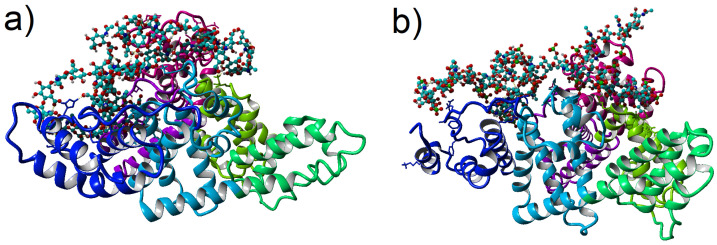
Structure of (**a**) HSA-HA, (**b**) HSA-CS6 complexes for highest affinity result in CaCl${}_{2}$ solution (solution is transparent on the picture). HSA is depicted as ribbon (bottom parts of picture), and its domains are colored as follows: IA-pink, IB-violet, IIA-light green, IIB-green, IIIA-light blue, and IIIB-blue. HA and CS6 are depicted as ball-stick (top parts of picture). Light blue atoms represent carbon, dark blue nitrogen, red oxygen, green sulfur and white hydrogen. Snapshots was taken using YASARA software after 100 ns MD simulations [19].

This study presents the analysis of the interactions between HSA and hyaluronic acid (HA)/ chondroitin-6-sulfate (CS6) in terms of the influence on conformations. As the topic is extensive, we are skipping here the conformation changes within albumin, focusing only on the GAGs. Such analysis of interaction between HSA and HA has been performed recently in [15]. HSA consists of a single chain of 585 amino acids, incorporating three homologous domains (I, II, and III) [16]. Domain I consists of residues 5–197, domain II includes residues 198–382, and domain III is formed from residues 383–569. Each domain comprises two sub-domains termed A and B (IA; residues 5–107, IB; residues 108–197, IIA; residues 198–296, IIB; residues 297–382, IIIA; residues 383–494, IIIB; residues 495–569), these are depicted in Figure 1. Some of the domains are more prone to binding to GAGs than others; however, the binding map can alter under some conditions of various diseases [17,18]. The binding mechanism is mainly due to ionic, hydrogen bonding and hydrophobic interactions [17].

GAGs are large complex carbohydrates. Depending on the monosaccharide types and the glycosidic bonds, GAGs can be divided into four groups: (i) hyaluronic acid, (ii) chondroitin sulfate, and dermatan sulfate (iii) heparan sulfate and heparin, and (iv) keratan sulfate. First, let us underline that HA is only a non-sulfated GAG. It is vital because sulfate groups in the GAG is one of the most crucial factors influencing the interaction map between a protein and the GAG [20]. Thus, two different examples of GAGs have been picked, which are essential components of synovial fluid, to study their conformational entropy while they are in the vicinity of the albumin: CS6 and HA. CS6 was shown to be an excellent material for bone regeneration as it is the main constituent of glycosaminoglycan in cartilage. CS6 is involved in bone homeostasis and in the coordination of osteoblastic cell attachment. Kim et al. investigated the role of chondroitin sulfate’s negative charge on the binding of cations (e.g., calcium and phosphate) and showed that the hydroxyapatite crystal formation was enhanced, accelerating osteogenesis.

The analysis of the interaction between HSA and HA or CS6 in the presence of various species such as water and ions is a meaningful task; as such, interaction is closely related to synovial systems’ unique properties [4]. The use of molecular modeling allows for researchers to evaluate the influence of various factors, such as the presence of ions and solvation on the properties of proteins, including their ability to bind ligands [21]. Following this, the present study aims to evaluate the effect of Na${}^{+}$, Mg${}^{2+}$, Ca${}^{2+}$ cations on the affinity of this two specific GAGs to HSA using: firstly docking, and secondly molecular dynamics (MD) methods. The studies are focused on the description of changes in conformation of the GAGs in the vicinity of the HSA.

GAGs are important complexes that participate in many biological processes through the regulation of specific proteins. Hence, their secondary structure and stability are very important to study. Both of above-mentioned properties can be well quantified by conformational entropy. As conformational entropy, we understand the Shannon entropy computed for bivariate histograms of chosen pairs of dihedral angles (see, Section 2.1) [22]. Variations of such entropy are considered to measure important properties of the biochemical processes [23,24]. In this research, we also refer to the informative interpretation of entropy. Basically, one can compute this entropy by studying the motion of the molecule. There are various methods dedicated to characterize these motions (e.g., NMR relaxation methods [25], AFM-unfolding [26], and neutron spectroscopy [27]). Theoretical studies based on classical mechanics approach are a reasonable alternative but computationally demanding [28,29]. The intermediate approach, we follow, is the computation of conformational entropy from all-atom MD simulations, see [23,30,31].

In more detail, conformation description of the GAGs relays on the analysis of their structures bound to HSA domains in aqueous ionic solution. This analysis is carried out to check whether there are any differences in the conformation of the glycosidic linkages between each oligosaccharide monomer of the GAG, when the kind of the ion is changed in aqueous solution. The linkages are investigated basing on specific dihedral angles. In the present paper, conformational entropy is computed from the frequency distribution of those angles’ values. We anticipate that those angles determine important characteristics, such as shape and stiffness. As the conformational entropy is calculated from the distribution of the angles, it is expected to be a crucial feature (enclosing the quantitative description in one numerical value).

## 2. Materials and Methods

We have performed all-atom simulations of the two model biosystems (one is HSA with HA and the other is HSA with CS6) in aqueous ionic solution. First, a molecular docking procedure has been executed to obtain preliminary information on the stability of the structure and to find the most energetically optimal places where each GAG attaches to the HSA. Next, energetically best-docked structures (sorted from the strongest connection to the weakest connected), with added water solution of chosen ions (Na${}^{+}$, Mg${}^{2+}$, Ca${}^{2+}$ and Cl${}^{-}$), have been subjected to MD simulations.

Chemical structures of HA and CS6 were obtained from Pubchem and modified to obtain chains of around 8 kDa (24 units). This modification relied on connecting units of selected GAGs until polymers of desired length were obtained. To acquire the most stable complexes, we docked GAG ligand (HA or CS6) to HSA using the VINA method [32] with their default parameters and point-charge force field [33] initially assigned according to the AMBER14 force field [34] (the HA molecule was parametrized by applying the GLYCAM06 force field [35]). Then, we damped the system to mimic the less polar Gasteiger charges used to optimize the AutoDock scoring function. The simulation was done with the YASARA molecular modeling program [19]. In each case (HA and CS6), 10 of the best distinctive complexes which differs in the position of GAG docked to HSA (best complexes means the complexes of the highest energy of binding and RMSD of complexes from the best binded complex values, which were computed by VINA) with −10 kcal/mol free energy of binding prepared for MD simulation.

MD simulations of HSA (PDB code: 1e78) with GAG have been run with YASARA software. Optimization of the hydrogen bonding network was included in the setup to increase the solute stability and a pK${}_{a}$ prediction (based on a Henderson–Hasselbalch equation) to fine-tune the protonation states of the protein residues at the given pH = 7.4 [36,37]. Optimization was done in three main steps: (a) pKa prediction was included to consider the influence of the pH on the hydrogen bonding network, (b) nonstandard amino acids and ligands were fully accounted for with the help of a chemical knowledge library in SMILES format, and (c) the use of the SCWRL algorithm allows for finding the globally optimal solution. In the case of the HSA-HA system, the complex has been immersed in one of the three aqueous 0.9% salt solutions NaCl, CaCl${}_{2}$ or MgCl${}_{2}$. In the case of HSA-CS6, it was 2% water solution of the same salts in pH = 7.0. After necessary minimization of the model system to remove clashes, the simulation was run for 100 ns using the AMBER14 force field [34] for the HSA, GLYCAM06 [35] for HA and CS6, and TIP3P for water. The van der Waals forces’ cut-off distance was set to 10 Å [38]. The particle Mesh Ewald algorithm was used for computing long-range interactions (e.g., electrostatic interactions) [39]. Simulations were performed under following conditions: temperature 310 K and pressure of 1 atm (NPT ensemble) [37]. Periodic boundary conditions were applied to a box of size roughly equal to 120 Å${}^{3}$. Berendsen barostat and thermostat were used to maintain constant pressure and temperature (relaxation time of 1 fs) [40]. The equations of motions were integrated with multiple time steps of 1.25 fs for bonded interactions and 2.5 fs for non-bonded interactions. In the considered simulations, the time step between saved states of the system equals 100 ps. Thus, the time series for 100 ns of simulations obtained 1000 save points. Snapshots of the two complexes after 100 ns of MD simulation have been presented in Figure 1.

All analyses and computation have been performed using YASARA and in-house written data processing programs in Python 3.8 [41].

### 2.1. Backbone Angles Determination

The method of entropy calculation, used in this study, relies on computation of the frequency distribution of the backbone’s dihedral angles (Φ,Ψ), usually presented in the form of a Ramachandran-type plot [31,42]. It provides a simplistic view of the conformation of a molecule by clustering angles (Φ,Ψ) into district regions (bear in mind that there are two sets of such pairs of angles, which will be discussed later).

**Figure 2:**
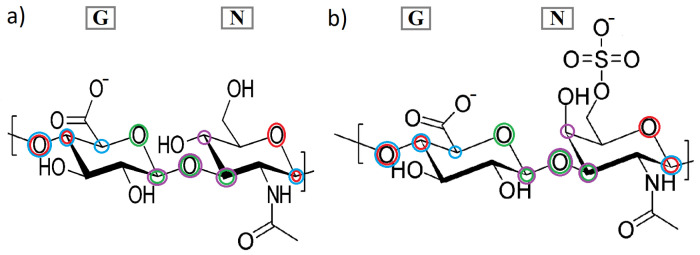
Structures of (**a**) HA and (**b**) CS6 with dihedral angles: $\Phi_{1-4}$—red circles, $\Psi_{1-4}$—blue circles (N-G linkage), $\Phi_{1-3}$—green circles, $\Psi_{1-3}$—violet circles (G-N linkage).

The CS6 consists of glucuronic acid (GlcA) and galactosamine (sulfated at C-6 atom of galactosamine; GalNAc), while HA consists of glucuronic acid (GlcA) and acetylglucosamine (GlcNAc). Linkages between the two monosaccharides are as follows: in the case of CS6, it is [4)-β-GlcA-(1⟶3)-β-GalNAc-)1⟶], and for HA: [4)-β-GlcA-(1⟶3)-β-GlcNAc-)1⟶] (see, Figure 2). Using abbreviation G for GlcA and N for GlcNAc (in HA) or GalNAc (in CS6), we can depict the glycosaminoglycans as linear heteropolysaccharide chains consisted of repeating disaccharide units [31]. In the presented study, the chains consist of NG units repeated 24 times. Two sets of torsion angles describe the conformations around the glycosidic linkages: $\Phi_{1-4}$ and $\Psi_{1-4}$ (N-G linkage), and $\Phi_{1-3}$ and $\Psi_{1-3}$ (G-N linkage) [31]. Thus, in the GAG chains, there are 24 of $\Phi_{1-4}$ and $\Psi_{1-4}$ angles, and 23 $\Phi_{1-3}$ and $\Psi_{1-3}$. These angles can be written as follows:

(1) $$\begin{array}{cc} \; & \Phi_{1-4}=O5(N)\text{-}C1(N)\text{-}O1(N)\text{-}C4(G),\\ & \Psi_{1-4}=C1(N)\text{-}O1(N)\text{-}C4(G)\text{-}C5(G),\\ & \Phi_{1-3}=O5(G)\text{-}C1(G)\text{-}O1(G)\text{-}C3(N),\\ & \Psi_{1-3}=\;C1(G)\text{-}O1(G)\text{-}C3(N)\text{-}C4(N). \end{array}$$

All dihedral angles in Equation (1) have been presented in Figure 2. The available conformational space of the GAGs’ chains depends mainly on the two torsion angles. As mentioned before, a goal of the present study is to investigate how various features of the system (ions, HA vs. CS6) affect the frequency distribution of Φ and Ψ torsion angles. As the informative feature of above-mentioned frequency distribution, conformational entropy has been used [24,31]. Note that, in information theory, entropy is the measure of the information tied directly to the variable (univariate or multivariate) [22,43]. Using this analogy, we can analyse which pairs of dihedral angles are more or less informative for specific GAGs and ions.

### 2.2. Entropy Calculation

**Figure 3:**
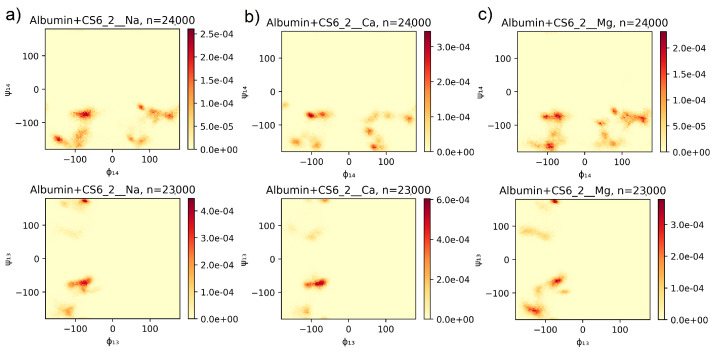
Normalized histograms of values of angles ($\Phi_{1-4}$, $\Psi_{1-4}$) (top) and ($\Phi_{1-3}$,$\Psi_{1-3}$) (bottom) for main chain CS6 with (**a**) Na${}^{+}$, (**b**) Ca${}^{2+}$, and (**c**) Mg${}^{2+}$. Angles were taken from the whole time series of the YASARA simulation. The symbol *n* is a number of angles’ pairs and is equal to number of angles type (24 for angles 1,4 and 23 for angles 1,3, cf. Section 2.1) multiplied by number of time points (1,000).

**Figure 4:**
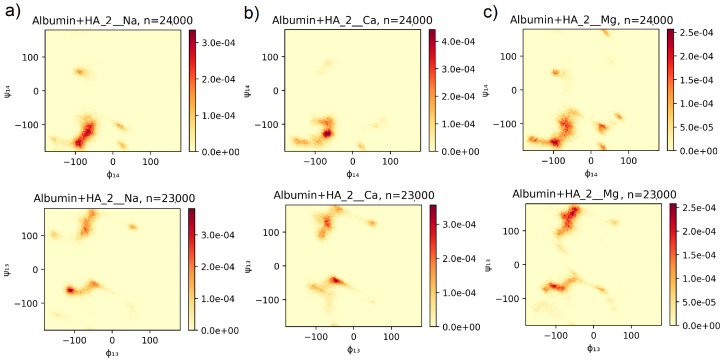
Normalized histograms of values of angles ($\Phi_{1-4}$, $\Psi_{1-4}$) (top) and ($\Phi_{1-3}$,$\Psi_{1-3}$) (bottom) for main chain HA with (**a**) Na${}^{+}$, (**b**) Ca${}^{2+}$, and (**c**) Mg${}^{2+}$. Angles were taken from the whole time series of the YASARA simulation. The symbol *n* is a number of angles’ pairs and is equal to number of angles type (24 for angles 1,4 and 23 for angles 1,3, cf. Section 2.1) multiplied by number of time points (1,000).

For each pair of subsequent mers of GAG’s chain, the time series (containing 1000 points) of the dihedral angles have been obtained. As described in Section 2.1, following pairs of these angles: $\Phi_{1-4}$ vs. $\Psi_{1-4}$ and $\Phi_{1-3}$ vs. $\Psi_{1-3}$ have been analyzed [31]. 2D histograms have been calculated from angles values of all subsequent pairs of mers (and all time steps). Histograms of the stronger bound structures (from the 10 picked as described in Section 2), for different ionic solutions, have been presented in Figure 3 for CS6 and in Figure 4 for HA.

Following an approach described in [44] (see Figure 8 therein), the conformation entropy has been computed from the 2D histograms of pairs of angles. In more detail, from each histogram, the Shannon entropy [22,23,30] has been computed using the formula:

(2) $$S=-R_{0}\sum_{i,j}p_{i,j}\log\left(p_{i,j}\right).$$

Here, $p_{i,j}$ is the empirical probability of the first angle being in the *i*th bin and the second angle being in the *j*th bin, and $R_{0}=8.314$ J·K${}^{-1}\cdot$mol${}^{-1}$ is the (scaling) gas constant.

## 3. Results

Some HSA segments are more prone to creating intermolecular interactions than others. The complexity of protein–GAG interactions is in part caused by the conformational flexibility of the GAG’s chain. An affinity of the HSA to GAGs, firstly tested by docking method, has been present in Table 1 sorted by binding energy. While the docking method relies on adjusting a ligand to a receptor in crystal form, then putting the complex into a water solution changes the intermolecular interactions map. After equilibration and 100 ns of MD simulations, the binding energy changed, and the order of best-bound complexes changed. In the case of CS6, the new order depended on added ions, and its value (averaged over three realizations’ binding energies with a different salt added) has been written in the first column of Table 1 in the brackets. HSA binding sites did not change much during the MD simulations. However, the number of interactions such as hydrophobic-polar, hydrogen bonds, ionic, and bridges have changed.

A closer analysis of the interactions for HSA-CS6 complexes will be the subject of another study, similar to the ones performed for HSA-HA complexes [15]. In the present paper, we are focused only on the conformational entropy of the GAGs chains. In the case of CS6, the most stable turned out realization was number 2, thus the situation when CS6 wrapped around the HSA and bound to IA-IB-IIA-IIIA-IIIB domains had the strongest binding to IIIA. No matter what ions have been added to the system, the binding energy stays high compared to the rest of the realizations (thus with different initial conditions of the binding map). After docking, amino acids that created the higher number of interactions with CS6 were Glu and Thr, and next in frequency: Lys and Asp. MD simulations show that Arg and Lys are more prone to create more ionic interactions and hydrogen bonds than the other amino acids due to their positive charge with negatively charged sulfate and carboxyl groups.

In the case of HA, a similar situation can be seen. The most stable was complex 2, i.e., composed of HSA’s domains IA-IB-IIIA-IIIB. The most binding amino acids, in general, were Thr, Glu and Lys. It is very important that domains IB, IIIA, and IIIB are key domains for the HSA transport function responsible for the heme-binding site (IB), Sudlow’s site II (IIIA), and thyroxine-binding site (IIIB) [45]. Comparing Table 1 to Table 2, one can notice that the HSA best binding segments differ slightly between HA and CS6 but are similar for the two first best-docked structures. In both cases, IIIA and IIIB domains clearly prevailed in creating the highest number of interactions between GAG and the protein. This is because domains IIIA and IIIB are domains that show a positive net charge on the surface that allows for binding with negatively charged GAGs. In addition, in both cases, a fragment of GAG’s chain strongly interacted with an IA domain. Analyzing differences between the values of energy of binding (averaged) in both cases, HA bound to albumin about 10% stronger than CS6 (see Table S1 in Supplementary Materials).

The method for computation of conformational entropy, based on a Ramachandran-type plot created for the pairs of dihedral angles (Φ,Ψ) [31,42], has been discussed in Section 2. Results of the computation have been presented in Figure 3 for the CS6 and in Figure 4 for the HA. Both of the results have been presented only for the best-bound complexes because the rest of the results had very similar characteristics. For comparison, three different realizations of YASARA simulations (thus, realizations with different initial structures) have been presented in Supplementary Materials Figures S1 and S2. The most probable angles, taken from Figure 3 and Figure 4, have been presented in Table 3.

In the case of CS6, ($\Phi_{1-4}$, $\Psi_{1-4}$) angles arranged in few clusters at ranges about: $-150^{\circ }$ – $-60^{\circ }$ for $\Phi_{1-4}$ and $-180^{\circ }$ – $-60^{\circ }$ for $\Psi_{1-4}$ with the highest probability of occupancy near two more narrow angle ranges with a maximum at $\left(-72^{\circ },-76^{\circ }\right)$ (cf. Figure 3, top line). There can also be seen, however, the second angle region of about: $50^{\circ }$–$180^{\circ }$ for $\Phi_{1-4}$ and $-180^{\circ }$ – $-50^{\circ }$ for $\Psi_{1-4}$, also with few narrowed clusters with a high probability of occupancy. The places of the spots and their intensity differ slightly between simulations with different ionic solution. In particular, in the case of Ca${}^{2+}$, most of the angles have been centered in one specific range around $\left(-104^{\circ },-76^{\circ }\right)$, while, in the cases of Mg${}^{2+}$ and Na${}^{+}$, more than one high probability place can be seen. In the case of Mg${}^{2+}$, the distribution of the angles is the most uniform but with, similar to the Na${}^{+}$ case, a maximum at $\left(-68^{\circ },-76^{\circ }\right)$.

The Ca${}^{2+}$ cations are distinguished by the fact that they form many more ionic interactions with the molecules than Na${}^{+}$ and Mg${}^{2+}$. This can be the reason for the differences in the ($\Phi_{1-4}$, $\Psi_{1-4}$) distribution plot. The greater probability cluster, which contains the maximum, is placed in the range of angles obtained lately in [46] for similar computer simulations of various kinds of chondroitin sulfate in water solution without any protein contribution. Thus, it can be clearly seen that the vicinity of HSA changed this crucial angle distribution, making them more disordered but still in a specific way. For proteins, similar (Φ, Ψ) angles are responsible for the formation of right-handed α-helices [42].

Despite the different chemical nature of protein and GAG molecules because peptide groups are linked at an α-carbon atom, not an oxygen like in the case of GAGs, the dihedral angles show how the chain-building units are rotated across the whole chain. Thus, the output secondary structure looks similar. In [46], there is, however, a lack of the second region on the angles, observed in our case with the positive values of $\Phi_{1-4}$ angles.

In the case of angles ($\Phi_{1-3}$,$\Psi_{1-3}$) for CS6, we can see quite different plots than the ones for ($\Phi_{1-4}$,$\Psi_{1-4}$) (see Figure 3 bottom line, and Figure S1 in Supplementary Materials). According to [46], the differences were expected because, for angles 1–3, the most probable occupancy should be in the region of $-100^{\circ }$–$-30^{\circ }$ (Φ) and $70^{\circ }$–$180^{\circ }$ (Ψ). In our results, the shadow (slightly visible red color on the plot) of those angles can be seen, especially in the case of Na${}^{+}$ and Mg${}^{2+}$, where the maximum is placed within this range (cf. Table 3), near $\left(-76^{\circ },169^{\circ }\right)$. Most of the angles have had the values similar like in the case of ($\Phi_{1-4}$,$\Psi_{1-4}$) but more focused on regions near $\left(-86^{\circ },-76^{\circ }\right)$ (maximum for Ca${}^{2+}$ case).

**Figure 5:**
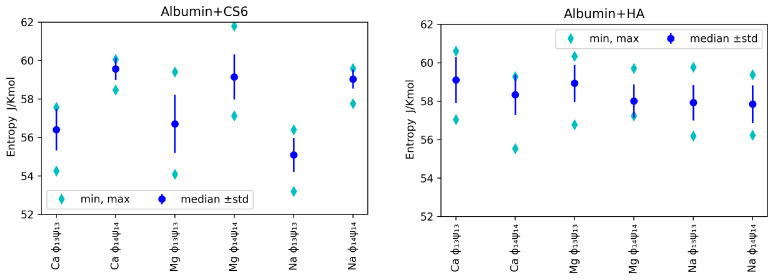
Median, maximal and minimal entropy (over N=10 realizations) for chosen ions and angles pairs for CS6 **(left panel)** and HA (**right panel**). As we have N=10 realizations, the minimal entropy can by considered as the estimate of the 10’th percentile (first quantile), and the maximal one as the estimate of the 90’th percentile (9’th quantile).

**Figure 6:**
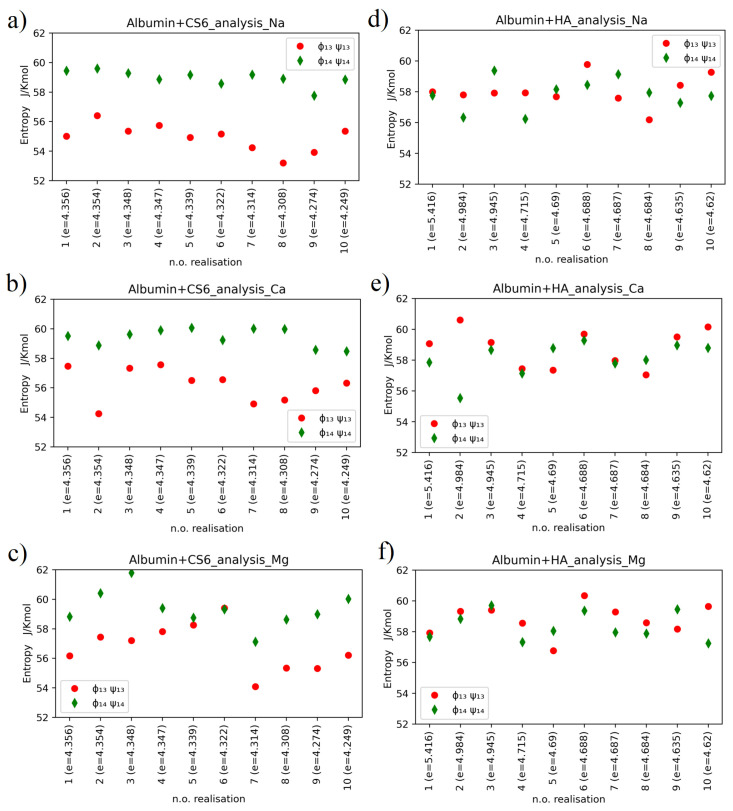
Conformational entropy for CS6 with (**a**) Na${}^{+}$, (**b**) Ca${}^{2+}$, and (**c**) Mg${}^{2+}$, and HA with (**d**) Na${}^{+}$, (**e**) Ca${}^{2+}$ and (**f**) Mg${}^{2+}$.

Conformational entropy for most cases is in the same range. However, there are noticeable differences in entropy between 1–3 and 1–4 angles. HA 1-4 angles show lower entropy i.e., are more stable than 1–3. The opposite is true for CS6. This can be explained by neighbouring groups. Carboxyl in HA and sulfur for CS6 form more stable contacts. Although acetyl group is highly reactive, it does not influence stability as dominant groups. The carboxyl group in CS6 is still weaker as compared to sulfur, which makes the contact more stable, as can be seen in Figure 5. The same behavior can be seen in Figure 6. The introduction of divalent ions increases entropy due to their destabilizing impact on protein, which is even more prominent for concentrations used in the present study.

Molecular conformational space available for HA chain in solution has been studied in [31]. The authors have searched for stable ordered forms of HA and have found many helices-type conformations (right- and left-hand side) that the HA chains prefer. Their findings based on potential energy computation for specific ($\Phi_{1-4}$,$\Psi_{1-4}$) and ($\Phi_{1-3}$,$\Psi_{1-3}$) angles are presented in Figure 2 of [31]. They obtain the location of three distinct regions with minimum potential energy surfaces. A main region consists of two wells (denoted as A–B in Figure 2 of [31] mentioned). Using the approach presented in this study, it is possible to compare the preferred dihedral angles for the HA chain, which is placed alone in the solution and in the vicinity of the HSA protein. In general, all these A-E regions (cf., Figure 2 in [31]) have been found in presented simulation results, but the intensity of these regions on the probability map varies depending on realizations (thus binding sites) and ion addition (see Figure 4 and Figure S2 in Supplementary Materials. In [31], for ($\Phi_{1-4}$,$\Psi_{1-4}$) angles, the main A–B region is placed in the area about $-120^{\circ }$–$-60^{\circ }$ for $\Phi_{1-4}$ and $-180^{\circ }$–$-100^{\circ }$ for $\Psi_{1-4}$, which is in accordance with results obtained in Figure 4 for the best docked HSA-HA complex. The probability of finding the angles in clusters A and B is almost equal in cases of realizations with the addition of Na${}^{+}$ and Mg${}^{2+}$, but, in the case of Ca${}^{2+}$, B prevails over A. The maximum has been found about $\left(-72^{\circ },-126^{\circ }\right)$ (B) and $\left(-97^{\circ },-155^{\circ }\right)$ (A). Moreover, a few different lighter clusters have been found similar to regions C, D and E.

In Figure 4, one can see two clearly identified clusters for ($\Phi_{1-3}$,$\Psi_{1-3}$) angles and two smaller ones. The first of the bigger cluster, with a maximum at about $\left(-115^{\circ },-61^{\circ }\right)$ in the case of Na${}^{+}$, suits region C in [31], and the second, near the maximum $\left(-54^{\circ },151^{\circ }\right)$ in the case of Mg${}^{2+}$, is placed in region A overflowing to region B. Region D, about $\left(50^{\circ },120^{\circ }\right)$ angles, is also present.

Median, minimal and maximal values (over realizations) of entropy for various ions, angles and GAGs are presented in Figure 5. Bear in mind that, as we used N=10 realizations, the minimal value can be used to roughly estimate first quantile, while the maximal value to roughly estimate the 9’th quantile.

In Figure 5, one can observe that, for analysed angles, ions and GAG type, entropy was in general greater for ($\Phi_{1-4}$,$\Psi_{1-4}$) angles than ($\Phi_{1-3}$,$\Psi_{1-3}$) in the case of CS6, but it was the opposite situation for HA: the entropy was slightly greater for ($\Phi_{1-3}$,$\Psi_{1-3}$). As lower entropy shows lesser disorder in the system, the most stable systems were those with Na${}^{+}$ ions added in both cases: HA and CS6 (cf., Figure 5). Complexes with Ca${}^{2+}$ ions usually have had slightly higher entropy. Referring to the informative interpretation of entropy, one can conclude that, in the case of CS6, the pair ($\Phi_{1-3}$,$\Psi_{1-3}$) carries significantly less information of the system than the pair ($\Phi_{1-4}$,$\Psi_{1-4}$). This is not the case for HA. The difference is related to the presence of the sulfate group in GalNAc in CS6 that is more prone to forming hydrogen bonds and ionic contacts with HSA.

Entropy values for CS6 and HA with different ions, taken separately for each of computer experiment realizations, have been presented in Figure 6.

Relatively large variations of the entropy between realizations are observed in the case of HSA-HA simulation results. Entropy varies within the range of 53–62 $\frac{J}{K\mathrm{mol}}$ for CS6 and within the range of 55–61 $\frac{J}{K\mathrm{mol}}$ for HA. Thermal noise, or some non-equilibrium processes, can have some effect on these variations, but also the different binding sites of the two molecules (that varies between realizations) are of big importance for the entropy behaviour. The variations of entropy values may also coincide with rather high estimation error of this value.

The hypothesis that entropy value is tied to the value of the binding energy between the protein and the GAG is not supported by our simulation results. In more detail, the smallest entropy value, roughly $53\frac{J}{K\mathrm{mol}}$, has been obtained for realization number 8 of the HSA-CS6 complex with Na${}^{+}$. This was the case, where only IA and IB domains of HSA were bound to CS6; thus, the protein did not affect the conformation of the GAG’s chain by deformation of the (Φ,Ψ) angles much. The highest entropy value has been reported for case 3 of the HSA-CS6 complex with Mg${}^{2+}$. Here, the binding site was very similar to the one with greater entropy (number 8): IA and IIA. Entropy records for realization number 2 of the HSA+HA complex with addition of Ca${}^{2+}$ are very interesting. In this realization, there were a huge difference between entropy for ($\Phi_{1-4}$,$\Psi_{1-4}$) angles and ($\Phi_{1-3}$,$\Psi_{1-3}$) angles. In this case, the HSA and HA molecules were best bound after MD simulation from all the realizations.

## 4. Discussion

A common pattern on the maps presented in Figure 4 and the ones presented in Figure 2 in [31] indicates that the proximity of the HSA protein does not prevent the HA chain from taking the shape of a helix (cf., Figure 5 in [31]). In the case of CS6, positions of preferred (Φ,Ψ) angles regions have been recently reported in [46]. The location of the angles presented in this study for HSA-CS6 complexes overlaps with those for the HA chain in [31]. Therefore, it can be concluded that CS6 keeps its α-helix structure. However, distributions of the (Φ,Ψ) angles (Figure 3 and Figure 4) show that GAGs can show helix structures and the random coil conformations at various ratios. Bear in mind that the frequency distributions of the (Φ,Ψ) angles are created from all NG units over the whole time; thus, we cannot say anything about how the conformations of the chains evolved. Instead, we see the static characteristic maps, which can tell us about the stability of the conformations in the simulation process.

We have demonstrated that conformational entropy is a parameter that enables us to characterize the structure of GAGs in interaction with HSA globally. We can see that it is slightly dependent on the pair of angles but rather for the CS6 case. In the case of CS6, the lower entropy value of the ($\Phi_{1-3}$,$\Psi_{1-3}$) angles compared to the entropy value of ($\Phi_{1-4}$,$\Psi_{1-4}$) angles indicate that mathematical features of the histograms for the angle pair 1-4 relative to the angle pair 1–3 must be more uniform. We understand this idea that ($\Phi_{1-4}$,$\Psi_{1-4}$) angles during simulations appear more uniform in the space of its value than angles ($\Phi_{1-3}$,$\Psi_{1-3}$) in the space of its value. This property is not visible for the angles in the case of HA.

Numerically derived histograms have various numbers of maxima, but these local maxima may be wider or narrower, which is essential in the entropy calculated here. Therefore, our work builds upon [46], which focuses on a sole CS6 and shows that a smaller number of regions are preferred to occupy by (Φ,Ψ) angles than our work that shows a larger number where HSA was added to the solution.

As the entropy is one of the features of above-mentioned histograms, we may expect other features of these histograms to be also informative about particular GAG, ion, or monomer. Hence, further analysis concerning pattern recognition machine learning techniques like SVM (Support Vector Machine) [47] and its modification for colored image processing [48] can be performed to extend the research. Another approach to this problem may be the colored image segmentation by a random walk [49], Sub-Markov random walk [50] or the Hurst Exponent image processing in [51,52]. Alternatively, one can analyze angles as multivariate series and process them with dedicated tools such as higher-order multivariate cumulants, see [53]. The goal of deep analysis mentioned above would be to search for more sensitive features and analyze whether entropy is a stable feature compared to others. If such features can be determined, their utility may appear in analytical, chemical medical applications. The secondary GAGs structure resembles flat bands transformed into a helix or twisted into a sheet originating from intermolecular hydrogen bonds. In diluted GAG solutions, the macromolecules have semi-rigid coiled chains and could form helix bands and even helical rings. Due to formation of a rigid helix, the macromolecules of GAG attract a great quantity of water and organize the broad domains of the tertiary polymer structure [54]. Binding to HSA reduces degrees of freedom of polymer and thus mechanical properties. On the other hand, HA and CS6 at higher concentrations, external force or other factors can change HSA’s tertiary structure, forming material of different properties.

## 5. Conclusions

Macromolecular complexes are building blocks in the functioning of physiological processes. When optimal conditions are fulfilled for given pairs, the system can function efficiently. Protein–ligand interactions are critical to optimal biochemical, biological, or biophysical results. Often, a given complex can serve several functions, as in the case presented in this study, where HA-HSA complexes decrease friction and can be used in drug delivery systems. Our results show how crucial components of synovial fluid interplay with each other at equilibrium. We have shown that HA and CS6 can form stable complexes with HSA.

Moreover, the binding sites for both molecules overlap, which indicates that they both can induce a similar effect on HSA while functioning. The molecular mass used in this study is one limitation of the presented results, as interactions (and mechanical and biological properties) between GAGs and proteins strongly depend on their molecular mass and concentration. This fact emerges from the chemistry of polymers of interest. GAGs chain’s amount of expansion is enormous for a semi-flexible polymer. The polymer configuration is constantly in a state of motion and change. However, the water increases the effective size of each hyaluronic acid because of its hydrophilic nature. The mass increase results in the average density decrease because the increase in mass is slower than in the volume. Thus, GAGs chains with a high molecular weight (more than 1,000 kDa) occupy a substantial volume. However, adsorption at HSA strongly influences the local mechanical properties of GAGs resulting in efficient lubrication.

## Figures and Tables

**Table 1:** Binding ranks of HSA-CS6 complexes. The first column contains: rank after docking (averaged rank after MD simulations). The strongest connected domains are marked in bold letters.

HSA-CS6 Complex Rank	HSA Binding Sites
1(5)	IA-IIA-IIIA-**IIIB**
2(1)	IA-IB-IIA-**IIIA**-IIIB
3(8)	IA-**IIA**
4(6)	IA-IIA-IIIA-**IIIB**
5(7)	IA-IIA-IIIA-**IIIB**
6(2)	IB-IIIA-**IIIB**
7(3)	**IA**-IIA-IIB-IIIA
8(10)	**IA**-IB
9(4)	IB-IIIA-**IIIB**
10(9)	IIA-**IIB**

**Table 2:** Binding ranks of HSA-HA complexes. The first column contains: rank after docking (averaged rank after MD simulations). The strongest connections are marked in bold letters.

HSA-HA Complex Rank	HSA Binding Sites
1(4)	IA-IB-IIIA-**IIIB**
2(1)	**IA**-IB-IIIA-IIIB
3(6)	**IA**-IB-IIIA-IIIB
4(10)	IIIA-**IIIB**
5(5)	IIB-**IIIA**-IIIB
6(8)	IA-IIIA-**IIIB**
7(2)	**IA**-IB-IIIA-IIIB
8(9)	IIIA-**IIIB**
9(7)	**IA**-IB-IIIA-IIIB
10(3)	**IIA**-IIB-IIIA

**Table 3:** Most frequent ($\Phi_{1-4}$, $\Psi_{1-4}$) and ($\Phi_{1-3}$, $\Psi_{1-3}$) angles for the best bound complexes of HSA-GAG.

CS6						
**n.o. Realization**	**Na${}^{+}$**		**Ca${}^{2+}$**		**Mg${}^{2+}$**	
2	$\Phi_{1-4}$	$\Psi_{1-4}$	$\Phi_{1-4}$	$\Psi_{1-4}$	$\Phi_{1-4}$	$\Psi_{1-4}$
	−72${}^{\circ }$	−76${}^{\circ }$	−104${}^{\circ }$	−76${}^{\circ }$	−68${}^{\circ }$	−76${}^{\circ }$
	$\Phi_{1-3}$	$\Psi_{1-3}$	$\Phi_{1-3}$	$\Psi_{1-3}$	$\Phi_{1-3}$	$\Psi_{1-3}$
	−76${}^{\circ }$	169${}^{\circ }$	−86${}^{\circ }$	−76${}^{\circ }$	−79${}^{\circ }$	173${}^{\circ }$
**HA**						
**n.o. Realization**	**Na${}^{+}$**		**Ca${}^{2+}$**		**Mg${}^{2+}$**	
2	$\Phi_{1-4}$	$\Psi_{1-4}$	$\Phi_{1-4}$	$\Psi_{1-4}$	$\Phi_{1-4}$	$\Psi_{1-4}$
	−72${}^{\circ }$	−126${}^{\circ }$	−68${}^{\circ }$	−126${}^{\circ }$	−97${}^{\circ }$	−155${}^{\circ }$
	$\Phi_{1-3}$	$\Psi_{1-3}$	$\Phi_{1-3}$	$\Psi_{1-3}$	$\Phi_{1-3}$	$\Psi_{1-3}$
	−115${}^{\circ }$	−61${}^{\circ }$	−47${}^{\circ }$	−47${}^{\circ }$	−54${}^{\circ }$	151${}^{\circ }$

## Data Availability

Data and processing code is available in the public repository at [41], https://github.com/iitis/polymer_entropy accessed on 7 June 2022.

## Supplementary Materials

The following supporting information can be downloaded at: https://www.mdpi.com/article/10.3390/e24060811/s1, Figure S1: Normalized histograms for different realizations for HSA-CS6 complexes.; Figure S2: Normalized histograms for different realizations for HSA-HA complexes.; Table S1: Binding energies in kcal/mol for 10 realizations of HSA-CS6 and HSA-HA complexes after docking.

## Abbreviations

The following abbreviations are used in this manuscript:

HSA	human serum albumin
HA	hyaluronic acid
CS6	chondroitin 6 sulfate
GAG	glycosaminoglycan
MD	molecular dynamics
PDB	protein data bank

## References

[1] Klein J., Raviv U., Perkin S., Kampf N., Chai L., Giasson S. (2004). Fluidity of water and of hydrated ions confined between solid surfaces to molecularly thin films. J. Phys. Condens. Matter.

[2] Gadomski A., Pawlak Z., Oloyede A. (2008). Directed ion transport as virtual cause of some facilitated friction–lubrication mechanism prevailing in articular cartilage: A hypothesis. Tribol. Lett..

[3] Gadomski A., Bełdowski P., Rubí J.M., Urbaniak W., Augé W.K., Santamaría-Holek I., Pawlak Z. (2013). Some conceptual thoughts toward nanoscale oriented friction in a model of articular cartilage. Math. Biosci..

[4] Dėdinaitė A., Claesson P.M. (2017). Synergies in lubrication. Phys. Chem. Chem. Phys..

[5] Raj A., Wang M., Zander T., Wieland D.F., Liu X., An J., Garamus V.M., Willumeit-Römer R., Fielden M., Claesson P.M. (2017). Lubrication synergy: Mixture of hyaluronan and dipalmitoylphosphatidylcholine (DPPC) vesicles. J. Colloid Interface Sci..

[6] Klein J. (2006). Molecular mechanisms of synovial joint lubrication. Proc. Inst. Mech. Eng. Part J J. Eng. Tribol..

[7] Liu C., Wang M., An J., Thormann E., Dėdinaitė A. (2012). Hyaluronan and phospholipids in boundary lubrication. Soft Matter.

[8] Siódmiak J., Bełdowski P., Augé W., Ledziński D., Śmigiel S., Gadomski A. (2017). Molecular dynamic analysis of hyaluronic acid and phospholipid interaction in tribological surgical adjuvant design for osteoarthritis. Molecules.

[9] Ghosh S., Choudhury D., Das N.S., Pingguan-Murphy B. (2014). Tribological role of synovial fluid compositions on artificial joints—A systematic review of the last 10 years. Lubr. Sci..

[10] Boldt J. (2010). Use of albumin: An update. BJA Br. J. Anaesth..

[11] Moman R.N., Gupta N., Varacallo M. (2021). Physiology Albumin.

[12] Ghosh S., Choudhury D., Pingguan-Murphy B. (2016). Lubricating ability of albumin and globulin on artificial joint implants: A tribological perspective. Int. J. Surf. Sci. Eng..

[13] Nečas D., Sadecká K., Vrbka M., Galandáková A., Wimmer M., Gallo J., Hartl M. (2021). The effect of albumin and *γ*-globulin on synovial fluid lubrication: Implication for knee joint replacements. J. Mech. Behav. Biomed. Mater..

[14] Kubiak-Ossowska K., Jachimska B., Mulheran P.A. (2016). How Negatively Charged Proteins Adsorb to Negatively Charged Surfaces: A Molecular Dynamics Study of BSA Adsorption on Silica. J. Phys. Chem. B.

[15] Bełdowski P., Przybyłek M., Raczyński P., Dedinaite A., Górny K., Wieland F., Dendzik Z., Sionkowska A., Claesson P.M. (2021). Albumin–Hyaluronan Interactions: Influence of Ionic Composition Probed by Molecular Dynamics. Int. J. Mol. Sci..

[16] Guizado T.R.C. (2014). Analysis of the structure and dynamics of human serum albumin. J. Mol. Model..

[17] Shi D., Sheng A., Chi L. (2021). Glycosaminoglycan-Protein Interactions and Their Roles in Human Disease. Front. Mol. Biosci..

[18] Qiu H., Jin L., Chen J., Shi M., Shi F., Wang M., Li D., Xu X.S., Su X., Yin X. (2020). Comprehensive Glycomic Analysis Reveals That Human Serum Albumin Glycation Specifically Affects the Pharmacokinetics and Efficacy of Different Anticoagulant Drugs in Diabetes. Diabetes.

[19] Krieger E., Vriend G. (2014). YASARA View—Molecular graphics for all devices–From smartphones to workstations. Bioinformatics.

[20] Gandhi N.S., Mancera R.L. (2008). The Structure of Glycosaminoglycans and their Interactions with Proteins. Chem. Biol. Drug Des..

[21] Bełdowski P., Yuvan S., Dėdinaitė A., Claesson P.M., Pöschel T. (2019). Interactions of a short hyaluronan chain with a phospholipid membrane. Colloids Surf. B Biointerfaces.

[22] Ben-Naim A. (2011). Molecular Theory of Water and Aqueous Solutions.

[23] Baruah A., Rani P., Biswas P. (2015). Conformational entropy of intrinsically disordered proteins from amino acid triads. Sci. Rep..

[24] Kruszewska N., Bełdowski P., Domino K., Lambert K.D., Gadomski A. (2019). Investigating conformation changes and network formation of mucin in joints functioning in human locomotion. Multiscale (Loco)motion—Toward Its Active-Matter Addressing Physical Principles.

[25] Sapienza P.J., Lee A.L. (2010). Using NMR to study fast dynamics in proteins: Methods and applications. Curr. Opin. Pharmacol..

[26] Thompson J.B., Hansma H.G., Hansma P.K., Plaxco K.W. (2002). The backbone conformational entropy of protein folding: Experimental measures from Atomic Force Microscopy. J. Mol. Biol..

[27] Fitter J. (2003). A Measure of Conformational Entropy Change during Thermal Protein Unfolding Using Neutron Spectroscopy. Biophys. J..

[28] Meirovitch H., Cheluvaraja S., White R. (2009). Methods for calculating the entropy and free energy and their application to problems involving protein flexibility and ligand binding. Curr. Protein Pept. Sci..

[29] Bhattacharjee N., Biswas P. (2012). Are ambivalent *α*-helices entropically driven?. Protein Eng. Des. Sel. PEDS.

[30] Baxa M.C., Haddadian E.J., Jumper J.M., Freed K.F., Sosnick T.R. (2014). Loss of conformational entropy in protein folding calculated using realistic ensembles and its implications for NMR-based calculations. Proc. Natl. Acad. Sci. USA.

[31] Haxaire K., Braccini I., Milas M., Rinaudo M., Pérez S. (2000). Conformational behavior of hyaluronan in relation to its physical properties as probed by molecular modeling. Glycobiology.

[32] Trott O., Olson A.J. (2010). AutoDock Vina: Improving the speed and accuracy of docking with a new scoring function, efficient optimization, and multithreading. J. Comput. Chem..

[33] Duan Y., Wu C., Chowdhury S., Lee M.C., Xiong G., Zhang W., Yang R., Cieplak P., Luo R., Lee T. (2003). A point-charge force field for molecular mechanics simulations of proteins based on condensed-phase quantum mechanical calculations. J. Comput. Chem..

[34] Maier J.A., Martinez C., Kasavajhala K., Wickstrom L., Hauser K.E., Simmerling C. (2015). ff14SB: Improving the Accuracy of Protein Side Chain and Backbone Parameters from ff99SB. J. Chem. Theory Comput..

[35] Kirschner K.N., Yongye A.B., Tschampel S.M., González-Outeiriño J., Daniels C.R., Foley B.L., Woods R.J. (2008). GLYCAM06: A generalizable biomolecular force field. Carbohydrates. J. Comput. Chem..

[36] Krieger E., Dunbrack R.L., Hooft R.W., Krieger B. (2012). Assignment of protonation states in proteins and ligands: Combining PKA prediction with hydrogen bonding network optimization. Methods Mol. Biol..

[37] Krieger E., Vriend G. (2015). New ways to boost molecular dynamics simulations. J. Comput. Chem..

[38] Hornak V., Abel R., Okur A., Strockbine B., Roitberg A.E., Simmerling C. (2006). Comparison of multiple Amber force fields and development of improved protein backbone parameters. Proteins Struct..

[39] Essmann U., Perera L.E., Berkowitz M.L., Darden T.A., Lee H.C., Pedersen L.G. (1995). A smooth particle mesh Ewald method. J. Chem. Phys..

[40] Berendsen H.J.C., Postma J.P.M., van Gunsteren W.F., Dinola A., Haak J.R. (1984). Molecular dynamics with coupling to an external bath. J. Chem. Phys..

[41] Sionkowski P., Domino K., Kruszewska N. (2022). Polymer_Entropy. https://github.com/iitis/polymer_entropy.

[42] Ramachandran G., Ramakrishnan C., Sasisekharan V. (1963). Stereochemistry of polypeptide chain configurations. J. Mol. Biol..

[43] Shannon C.E. (2001). A mathematical theory of communication. ACM SIGMOBILE Mob. Comput. Commun. Rev..

[44] Weber P., Bełdowski P., Domino K., Ledziński D., Gadomski A. (2020). Changes of Conformation in Albumin with Temperature by Molecular Dynamics Simulations. Entropy.

[45] Fasano M., Curry S., Terreno E., Galliano M., Fanali G., Narciso P., Notari S., Ascenzi P. (2005). The extraordinary ligand binding properties of human serum albumin. IUBMB Life.

[46] Nagarajan B., Sankaranarayanan N.V., Desai U.R. (2022). In-depth molecular dynamics study of all possible chondroitin sulfate disaccharides reveals key insight into structural heterogeneity and dynamism. Biomolecules.

[47] Burges C.J. (1998). A tutorial on support vector machines for pattern recognition. Data Min. Knowl. Discov..

[48] Khemchandani R., Saigal P. (2015). Color image classification and retrieval through ternary decision structure based multi-category TWSVM. Neurocomputing.

[49] Grady L. (2006). Random walks for image segmentation. IEEE Trans. Pattern Anal. Mach. Intell..

[50] Dong X., Shen J., Shao L., Van Gool L. (2015). Sub-Markov random walk for image segmentation. IEEE Trans. Image Process..

[51] Blachowicz T., Ehrmann A., Domino K. (2016). Statistical analysis of digital images of periodic fibrous structures using generalized Hurst exponent distributions. Phys. A Stat. Mech. Appl..

[52] Blachowicz T., Domino K., Koruszowic M., Grzybowski J., Böhm T., Ehrmann A. (2021). Statistical analysis of nanofiber mat AFM images by Gray-scale-resolved Hurst exponent distributions. Appl. Sci..

[53] Domino K. (2020). Multivariate cumulants in outlier detection for financial data analysis. Phys. A Stat. Mech. Appl..

[54] Snetkov P., Zakharova K., Morozkina S., Olekhnovich R., Uspenskaya M. (2020). Hyaluronic Acid: The Influence of Molecular Weight on Structural, Physical, Physico-Chemical, and Degradable Properties of Biopolymer. Polymers.

