# Supplementary material for: Effect of Ion and Binding Site on the Conformation of Chosen Glycosaminoglycans at the Albumin Surface
Source: Entropy (Basel). 2022 Jun 10;24(6):811. doi: 10.3390/e24060811 (PMC9222412; doi:10.3390/e24060811)
Supplement: Supplementary file 1 [file entropy-24-00811-s001.zip › entropy-1744828-supplementary.pdf]

Article

Supplementary materials to: Effect of ion and binding site on the conformation of chosen glycosaminoglycans at the albumin surface

Piotr Sionkowski [1], Piotr Bełdowski [2,*], Natalia Kruszewska [2], Piotr Weber [3], Beata Marciniak [4] and Krzysztof Domino [1]

[1] Institute of Theoretical and Applied Informatics, Polish Academy of Sciences; piotr.sionkowski@gmail.com, kdomino@iitis.pl;
[2] Institute of Mathematics and Physics, Bydgoszcz University of Science and Technology, 85-796 Bydgoszcz, Poland; piotr.beldowski@pbs.edu.pl, nkruszewska@pbs.edu.pl;
[3] Gdańsk University of Technology, G. Narutowicza 11/12, 80-233 Gdańsk; piotr.weber@pg.edu.pl;
[4] Faculty of Telecommunications, Computer Science and Electrical Engineering, Bydgoszcz University of Science and Technology, 85-796 Bydgoszcz, Poland; beata.marciniak@pbs.edu.pl;
* Correspondence: piotr.beldowski@pbs.edu.pl

Table S1. Binding energies in kcal/mol for 10 realizations of HSA-CS6 and HSA-HA complexes after docking.

n.o.	CS6	HA
1	4.356	5.416
2	4.354	4.984
3	4.348	4.945
4	4.347	4.715
5	4.339	4.69
6	4.322	4.688
7	4.314	4.687
8	4.308	4.684
9	4.274	4.635
10	4.249	4.62

Citation: Sionkowski, P.; Bełdowski, P.; Kruszewska, N.;Weber, P.; Marciniak, B.; Domino, K. Effect of Ion and Binding Site on the Conformation of Chosen Glycosaminoglycans at the Albumin Surface. Entropy 2022, 24, 811. https://doi.org/10.3390/e24060811

Received: 11 May 2022
Accepted: 8 June 2022
Published: 10 June 2022

Publisher's Note: MDPI stays neutral with regard to jurisdictional claims in published maps and institutional affiliations.

Copyright: © 2022 by the authors. Submitted to *Entropy* for possible open access publication under the terms and conditions of the Creative Commons Attri- bution (CC BY) license (https://creativecommons.org/licenses/by/4.0/).

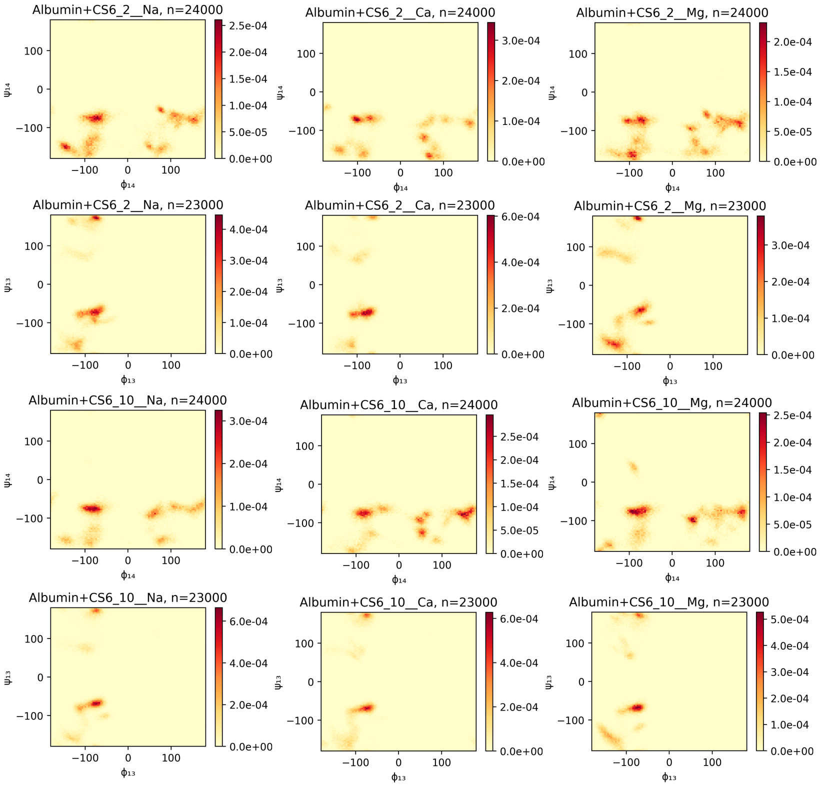

Figure S1. Normalized histograms for different realizations for HSA-CS6 complexes.

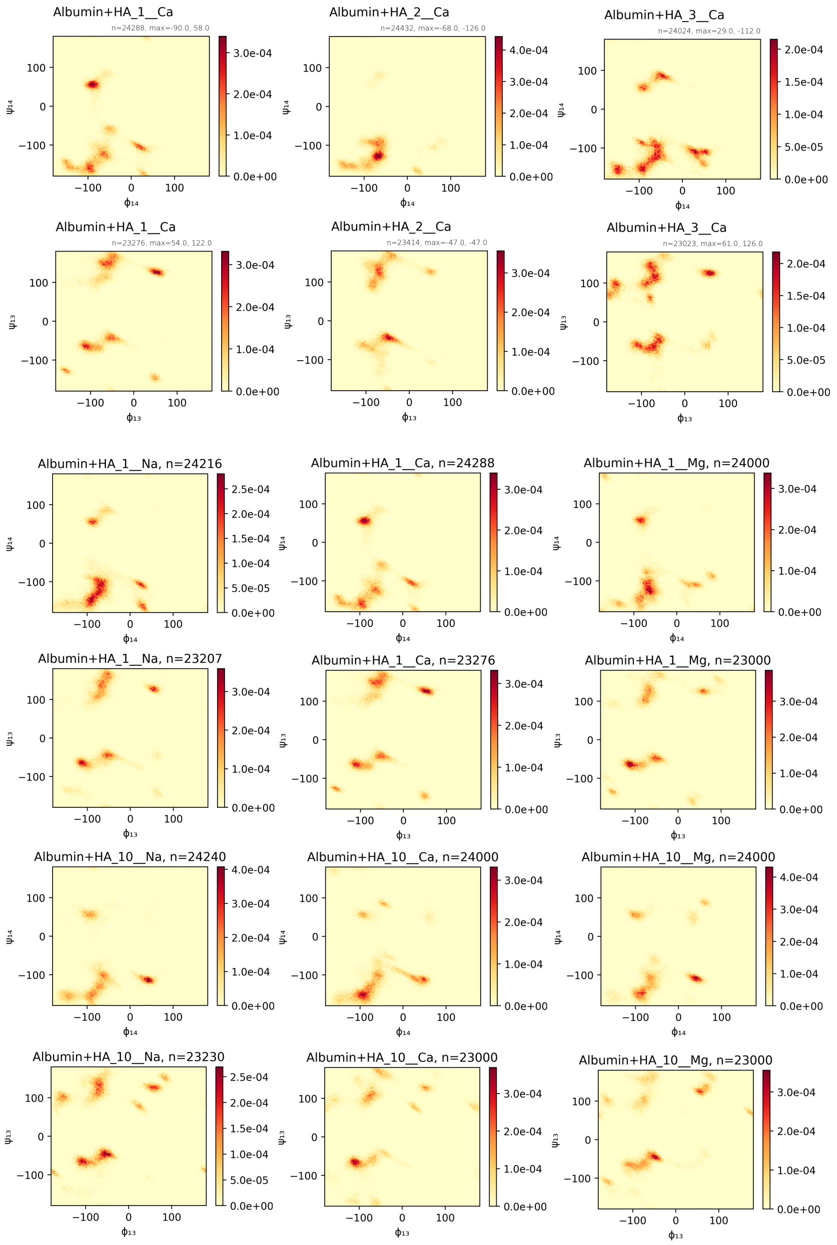

Figure S2. Normalized histograms for different realizations for HSA-HA complexes.
